# Leptomeningeal neuraxis relapse in glioblastoma is an uncommon but not rare event associated with poor outcome

**DOI:** 10.1186/s12883-023-03378-1

**Published:** 2023-09-15

**Authors:** Eric Wegener, Patrick Horsley, Helen Wheeler, Dasantha Jayamanne, Marina Kastelan, Linxin Guo, Chris Brown, Michael Back

**Affiliations:** 1 Department of Radiation Oncology Northern Sydney Cancer Centre, Royal North Shore Hospital, St Leonards Sydney, NSW 2065 Australia; 2 The Brain Cancer group, St Leonards, NSW Australia; 3 Sydney Medical School, University of Sydney, Sydney, Australia; 4 NHMRC Clinical Trials Centre, Sydney, Australia; 5 Genesis Cancer Care, Sydney, Australia; 6 Central Coast Cancer Centre, Gosford Hospital, Gosford, Australia

**Keywords:** Glioblastoma, leptomeningeal, LMD

## Abstract

**Background:**

Spinal neuraxis leptomeningeal metastasis (LM) relapse in glioblastoma is an uncommon event that is challenging to manage. This study aims to determine the incidence, associated factors, and outcome of LM relapse in patients with glioblastoma managed with radical intent.

**Methods:**

Patients managed for glioblastoma using the EORTC-NCIC (Stupp) Protocol from 2007 to 2019 were entered into a prospective ethics-approved database. Follow-up included routine cranial MRI surveillance with further imaging as clinically indicated. LM relapse was determined by MRI findings and/or cerebrospinal fluid analysis. The chi-square test of independence was used to evaluate clinico-pathologic factors associated with increased risk of subsequent LM relapse. Median survival post-LM relapse was calculated using Kaplan-Meier technique.

**Results:**

Four-hundred-and-seven patients were eligible, with median follow-up of 60 months for surviving patients. Eleven (2.7%) had LM at first relapse and in total 21 (5.1%) experienced LM in the entire follow-up period. Sites of LM relapse were 8 (38%) focal spinal, 2 (10%) focal brainstem medulla and 11 (52%) diffuse spinal. Median overall survival from initial diagnosis for the entire cohort was 17.6 months (95% CI 16.7–19.0). Median survival from LM relapse to death was 39 days (95% CI: 19–107). Factors associated with LM relapse were age less than 50 years (p < 0.01), initial disease located in the temporal lobe (p < 0.01) and tumours lacking MGMT promoter methylation (p < 0.01).

**Conclusions:**

LM relapse is an uncommon but not rare event in patients managed radically for glioblastoma. It is associated with poor outcome with the majority of patients deceased within two months of recognition.

## Introduction

Glioblastoma (GBM) is the most common primary brain tumour in adults. Despite aggressive therapy which may include surgical resection and adjuvant chemoradiotherapy, relapse is almost universal. Although there have been incremental improvements in survival since the introduction of temozolomide, still less than 10% of patients are alive more than 5 years following diagnosis [1, 2]. Relapse is most common local, at or near to the site of initial enhancing disease [3]. However a substantial minority (20–25%) relapse distantly within the brain (often defined as > 2 cm from the gadolinium-enhancing component on MRI at diagnosis) [3–5]. Distant in-brain relapse is associated with better overall survival from diagnosis than local relapse [4], possibly as it implies better local control and is associated with longer time to first progression. However, less well-recognised is a smaller and distinct sub-group of patients who develop leptomeningeal dissemination (LMD) and experience a dismal prognosis.

The body of literature addressing LMD in glioblastoma to date remains relatively limited. The symptomatic burden of LMD may include cranial nerve palsies, focal neurologic deficits, raised intracranial pressure, hydrocephalus, meningism and confusion, but LMD may also be asymptomatic in a substantial subgroup [6]. Few studies have systematically addressed risk factors for LMD, but possible associations have been made with patient (e.g. younger age [7]), tumour (e.g. anatomic location [8, 9], molecular features [10, 11]) and treatment (e.g. ventricle entry during craniotomy [12, 13]) factors. Several therapeutic options have been explored including systemic and intrathecal chemotherapy (particularly methotrexate [7] and cytarabine [14]), anti-angiogenic therapy (e.g. bevacizumab) [15], targeted therapy (e.g. BRAF inhibitors [11]) and radiotherapy [16], but all with limited success. Survival after the detection of leptomeningeal relapse is poor.

A better understanding of this condition and increased clinician awareness is a starting point to improve recognition, anti-cancer management and supportive care which may ultimately translate to better survival and quality of life. In this study, we aim to address the incidence, risk factors and prognosis of LMD within a large consecutive series of patients with glioblastoma managed initially with radical intent.

## Methods

### Patient population

A retrospective analysis was performed on adult patients managed with GBM through the Northern Sydney Cancer Centre Neuro-oncology MDT from 2007 to 2019. Patient-, tumour- and treatment-related data were entered into a prospective database, approved by Institutional Ethics Review Board. Eligible patients were those with histopathological confirmation of GBM and managed with definitive or adjuvant chemo-radiotherapy consistent with the EORTC-NCIC using the EORTC-NCIC (Stupp) Protocol [1]. Patients managed with hypofractionated radiotherapy (40 Gy in 15 fractions) were not included in the study analysis.

### Neurosurgical management

Patients were referred from multiple neurosurgical units. There was emphasis on maximal resection, however there was no uniform policy addressing extent of resection. Aggressive resections were enabled by use of techniques such as endoscopic surgery and awake craniotomy. In the absence of contraindications, 3T gadolinium enhanced MRI scans were performed preoperatively and postoperatively. Timing of the postoperative MRI varied, but generally was performed within first 48 h following resection. Metabolic imaging with FET/FDG scans were employed for patients with MRI demonstrating multifocal areas of enhancement or a suspicious region of either T2-FLAIR hyperintensity or non-enhancing T1 hypodensity. Pre- and first post-operative MRI scans were reviewed by a radiation oncologist with extensive neuro-oncology experience to visually estimate the extent of resection of the gadolinium-enhancing component of the tumour. This was classified as biopsy (< 50% of gadolinium-enhancing tumour excised), subtotal (50–90% excised) and near-total resection (> 90% excised).

### Neuropathological features

All patients had the diagnosis of GBM (WHO Grade IV) confirmed by standard immunohistochemical techniques. Proliferation index was obtained with Ki67% in the majority of patients. Molecular analysis including for isocitrate dehydrogenase (IDH) mutation, O^6^-methylguanine-DNA methyltransferase (MGMT) promoter methylation status and EGFR amplification was gradually introduced into practice from 2012 as evidence for their utility strengthened. Tumours diagnosed prior to and after 2016 were classified according to the WHO 2007 and WHO 2016 classification of CNS tumours respectively.

### Radiation therapy

Patients were treated with the dose fractionation schedule as per the EORTC-NCIC Protocol [1]. 60 Gy was delivered in 30 fractions over a six-week period initiating between day 21 to 28 post craniotomy. All patients received intensity modulated radiation therapy (IMRT) with image guided radiation therapy (IGRT).

### Systemic therapy

The initial systemic management followed the EORTC-NCIC Protocol Regimen with TMZ used in two phases: initially 75 mg/m^2^ daily during the RT, followed by a 4-week break; then 150-200 mg/m^2^ for days 1–5 every 28 days for 6–12 months [1].

During the study time period, patients may have been enrolled in multicentre adjuvant therapy clinical trials, and these studies may have provided additional treatments to the standard TMZ therapy. Only one study altered the standard TMZ regimen, the randomised Phase II VERTU trial [17] (see below).

### Clinical trial enrolment

A subset of patients were enrolled in clinical trials and were treated in accordance with the respective trial protocol.

Twenty-five patients were enrolled in the VERTU phase II trial [17], in which patients were randomised to receive radiotherapy with concurrent with the PARP inhibitor veliparib (200 mg twice daily) followed by adjuvant veliparib (400 mg twice daily, days 1–7 of a 28 day cycle for 6 months) and temozolomide versus standard chemoradiotherapy and adjuvant temozolomide as per the EORTC-NCIC (Stupp) protocol.

Twenty-one patients were enrolled in AVAglio (BO21990) [18] in which they were randomised to receive bevacizumab versus placebo. The investigational arm received bevacizumab concurrent with radiotherapy and temozolomide (10 mg/kg 2-weekly for 6 weeks), maintenance bevacizumab (10 mg/kg 2-weekly for 24 weeks) with temozolomide and then bevacizumab monotherapy (15 mg/kg every 3 weeks until progression or unacceptable toxicity).

Twenty-one patients were enrolled in ExCENTRIC [19] in which all patients received cilengitide 200 mg IV twice weekly from 1 week prior to chemoradiotherapy which was also combined with concurrent daily temozolomide and procarbazine (50 or 100 mg). Adjuvant therapy consisted of 6 cycles of temozolomide (50–60 mg) and procarbazine (50 or 100 mg) on days 1–20 every 28 days. Cilengitide was also continued for up to 12 months.

Nine patients were enrolled in CHECKMATE-143 [20] in which patients with glioblastoma at first relapse were randomised to either nivolumab (3 mg/kg) or bevacizumab (10 mg/kg) every 2 weeks until disease progression, unacceptable toxicity or death.

Six patients were enrolled in INTELLANCE-2 [21], a 4-arm study in which patients at or before first progression after adjuvant therapy were randomised to receive the experimental drug depatuxizumab mafodotin (ABT414) (1.25 mg/kg or 1.0 mg/kg every 2 weeks) alone, in combination with temozolomide, temozolomide alone, or lomustine alone.

Five patients were enrolled in CENTRIC (EORTC 26,071 − 22,072) [22] in which they were randomised to receive temozolomide chemoradiotherapy with or without cilengitide 2000 mg intravenously twice weekly. Both groups received 6 months of adjuvant temozolomide and the cilengitide group also received adjuvant cilengitide for up to 18 months.

Three patients were enrolled in ACT IV [23] in which patients with EGFRvIII expression who had completed adjuvant chemoradiotherapy were randomised to receive (in addition to standard adjuvant temozolomide) rindopepimut (500 microg mixed with 150 microg GM-CSF) or control (100 microg keyhole limpet hemocyanin) via monthly intraepidermal injection until progression or intolerance.

### Follow-up

**Fig. 1:**
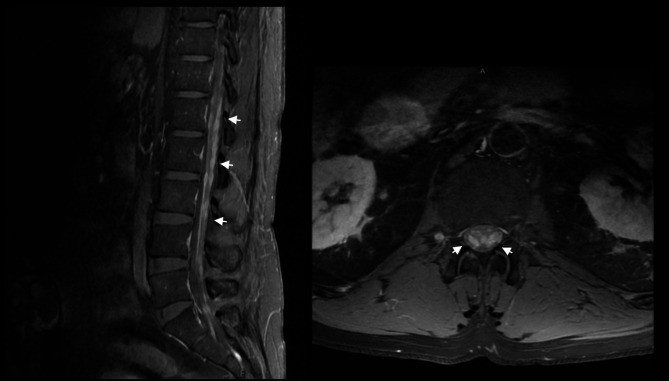
Sagittal (left) and axial (right) T1-weighted post-gadolinium magnetic resonance imaging sequences demonstrating prominent nodular leptomeningeal deposits (arrows) surrounding the conus medullaris and involving the cauda equina nerve roots

All patients were followed up closely with imaging. Baseline MRI occurred one month post RT then second monthly MRIs until completion of adjuvant temozolomide. After this three-monthly MRIs until end of year 3 post RT. From the fourth year after RT, MRI surveillance was continued at four to six month intervals until progression. Progression was confirmed according to the Response Assessment in Neuro-Oncology Working Group [24]. Additional imaging was obtained as clinically indicated. Features suggestive for pseudoprogression were actively investigated to exclude true relapse, including using FDG and FET-PET scans and short-interval serial MRI. Post-therapy outcome was recorded into the prospective database including site of relapse. Presence of spinal neuraxis LMD relapse was defined as surface or nodular enhancement occurring at any site from brainstem medulla to cauda equina. The vast majority of LMD was detected symptomatically. An example is given in Fig. 1. Of the symptomatic patients, 57% had back or neck pain and 30% presented with cranial nerve impingement symptoms. The remaining 13% of symptomatic patients presented with other varied neurological symptoms.

### Salvage therapy

Management at time of relapse was individualised based on performance status, disease extent, symptoms and prior therapy. This included the use of repeat craniotomy, second and third line cytotoxic chemotherapy, bevacizumab and re-irradiation. A spinal canal gadolinium enhanced MRI was performed if there was evidence of subependymal ventricular enhancement, leptomeningeal enhancement or symptoms suggestive of potential spinal dissemination (unusual radicular pain, urinary disturbance or limb weakness not consistent with intracranial disease). Cerebrospinal fluid confirmation was not required for diagnosis, but performed in the case of uncertainty regarding diagnosis of LMD on radiological findings.

### Statistical considerations

Overall survival time in months was calculated from the time of initial surgical diagnosis of WHO Grade IV glioma to death or close-out date on 1st January 2020. Survival time from date of LMD diagnosis in days was calculated from date of scan confirming LMD to death or close-out date on 1st January 2020. Survival as a function of time was plotted and analysed using the Kaplan-Meier method. Cox proportional hazards model was used to analyse the risk of death after LMD relapse. Univariate potential factors for LMD development were evaluated using a log rank test. All reported p-values are two-tailed. Statistical examination was done using commercial software (IBM SPSS Statistics, version 23, Armonk, NY, USA). The results were considered significant if p < 0.05.

## Results

### Patient characteristics

407 patients diagnosed with GBM and subsequently managed with IMRT and TMZ following the EORTC-NCIC Protocol between March 1st 2007 and September 30th 2019 were identified from the database and included in the analysis. The close-out date was 1st January 2020. There were seven patients who had less than 6 months follow-up. The median follow-up of all alive patients was 60 months. Patient and tumour characteristics are summarized in Table 1.

**Table 1:** Patient and tumour characteristics at initial management

	All patients (N = 407)	Patients with LMD relapse (N = 21)
Age at diagnosis		
< 50 years, n (%) ≥ 50 years, n (%) Median (years)	102 (25%) 305 (75%) 58	13 (62%) 8 (38%) 48
Tumour site, n (%)		
Temporal Frontal Parietal Occipital Thalamic Other	136 (33%) 113 (28%) 97 (24%) 34 (8%) 19 (5%) 8 (2%)	13 (62%) 2 (9.5%) 1 (5%) 2 (9.5%) 3 (14%) 0 (0%)
Extent of resection, n (%)		
Near-Total Subtotal Biopsy	177 (43%) 171 (42%) 59 (15%)	12 (57%) 7 (33%) 2 (10%)
Ki67 index, n (%)		
< 30% ≥ 30% Unknown Median	147 (36%) 219 (54%) 41 (10%) 30	5 (24%) 13 (62%) 3 (14%) 45
IDH1 mutation, n (%)		
Yes No	18 (4%) 389 (96%)	2 (10%) 19 (90%)
MGMT methylation, n (%)		
No Yes Unknown	123 (30%) 102 (25%) 182 (45%)	12 (57%) 0 9 (43%)
ECOG performance status at baseline, n (%)		
0 1 2 3,4	118 (29%) 172 (42%) 87 (21%) 30 (7%)	11 (52%) 7 (33%) 2 (10%) 1 (5%)

LMD: Leptomeningeal disease, IDH: Isocitrate dehydrogenase, MGMT: O^6^-methylguanine-DNA methyltransferase, ECOG: Eastern Cooperative Oncology Group

The median age for patients at diagnosis was 58 years with 25% of patients aged younger than 50 years. ECOG Performance status at initiation of RT was 0–1 and 2–3 in 71% and 29% of patients respectively. The two most frequent neuroanatomical sites were temporal lobe (33%) and frontal lobe (28%). Near total surgical resection was achieved in 43.5%, subtotal in 42% and 14.5% had biopsy. MGMT promoter methylation was performed in 55% of patients and methylation status was positive in 45% of those patients tested.

### Progression

Radiological or histopathological features suggestive for progression were noted in 334 of the 407 patients, with a median time from date of diagnosis to date of progression of 10.5 months (range = 1.4–81.1). Twenty one (5.1%) of the 407 patients were diagnosed with LMD relapse. Eleven (2.7%) of the 407 patients were diagnosed with LMD relapse at time of first progression. The median time from date of diagnosis to date of LMD relapse was 7.4 months (range = 1.6–48.7). Sites of LMD relapse included 8 (38%) focal spinal, 2 (10%) focal brainstem medulla and 11 (52%) diffuse spinal relapses. Two thirds of LMD neuraxis relapses occurred concurrently or subsequently with intracranial local relapse. Eight of 21 patients who had LMD relapse were enrolled on clinical trials, including 3 on VERTU, 3 on ExCENTRIC, 1 on ACT IV and 1 on AVAglio.

### Management of LMD Relapse

Management at time of leptomeningeal relapse was individualised based on patient performance status, symptoms and prior therapy. Of the 21 patients who developed LMD relapse, 7 received palliative systemic chemotherapy, of whom 3 also received bevacizumab and 2 received palliative radiotherapy. 2 further patients received palliative radiotherapy without chemotherapy, one of whom was also treated with bevacizumab. The remaining 12 patients were managed with best supportive care alone.

### Survival

**Fig. 2:**
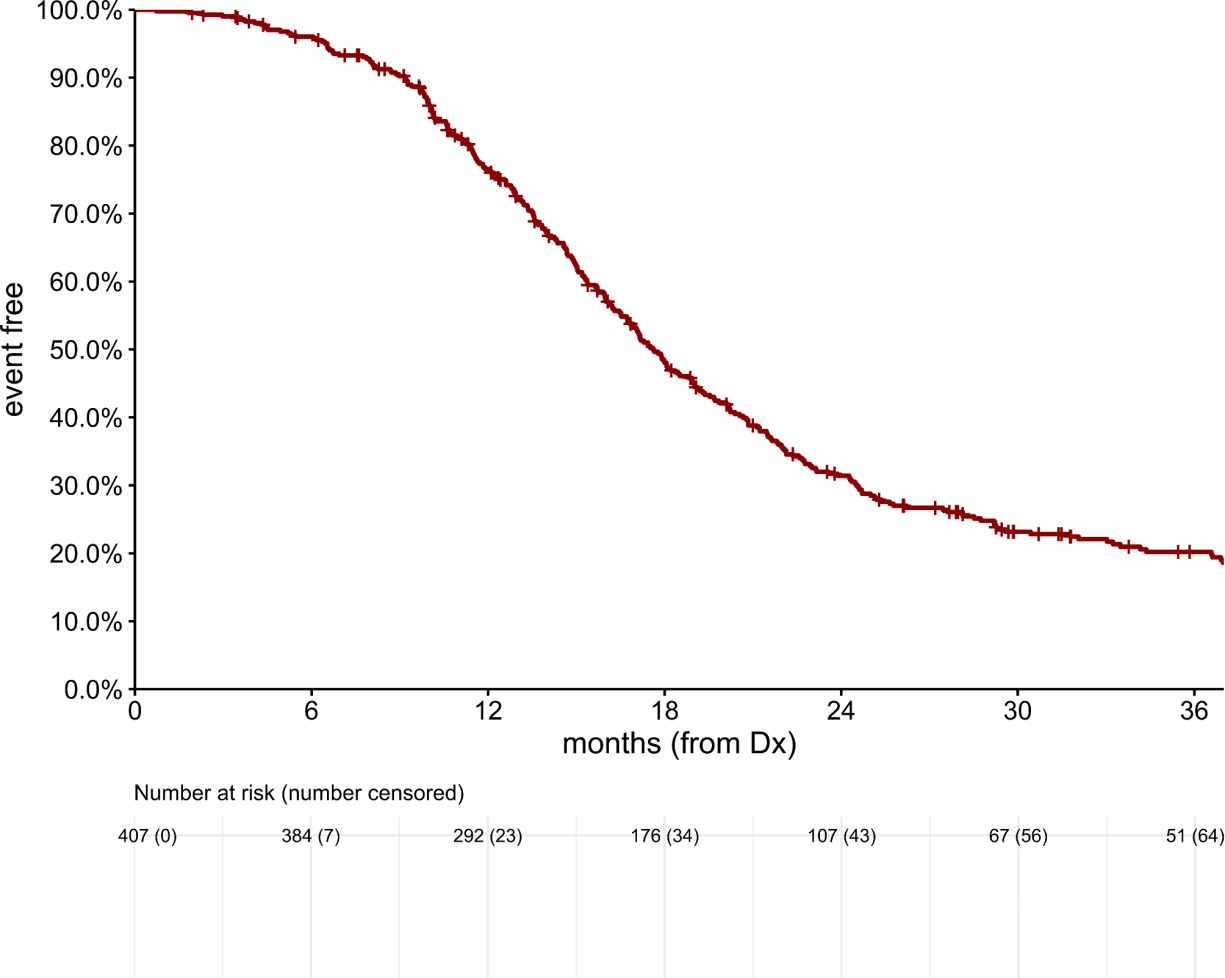
Overall survival from date of diagnosis. Median survival 9.2 months for LMD relapse cohort (95% CI: 7.9, 19.8) and 17.6 months for non LMD relapse cohort (95% CI: 16.7, 19.0)

The median survival from date of initial diagnosis for patients who experienced LMD relapse was 9.2 months (95% CI: 7.9, 19.8) as demonstrated in Fig. 2. Median overall survival for the entire cohort was 17.6 months (95% CI: 16.7, 19.0).

**Fig. 3:**
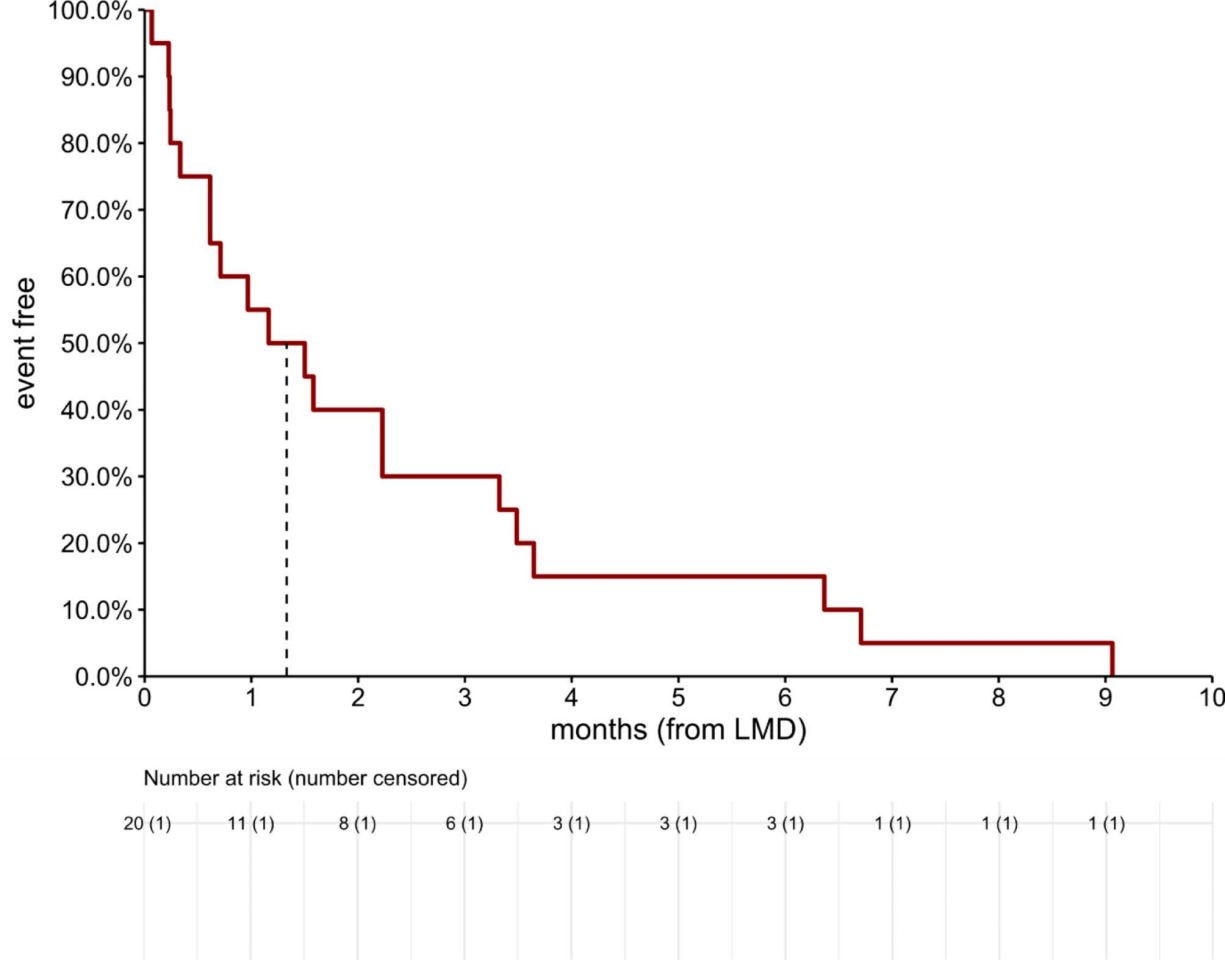
Overall survival from date of LMD relapse. Median survival 39 days (95% CI: 19, 107)

Following date of LMD relapse the median survival was only 39 days (95% CI: 19, 107), demonstrated in Fig. 3. Those patients who were diagnosed with LMD relapse were at an increased risk of death by a factor of 9.5 (95% CI: 6, 15).

Four patients who survived for more than 4 months post spinal LMD relapse had predominantly focal spinal disease; all were managed with systemic therapy and three had focal radiation therapy.

### Factors associated with LMD relapse

Factors associated with LMD relapse included patients age less than 50 years (p < 0.01), temporal lobe site of initial tumour (p < 0.01) and MGMT unmethylated status (p < 0.01) as detailed in Table 2. The median age at diagnosis of GBM for patients who experienced LMD relapse was 48 years compared to 58 years for the whole cohort. Patients who had LMD relapse had a median Ki67 index of 45% compared to 30%. However on univariate analysis, Ki67 index ≥ 30% compared to < 30% was not significantly associated with LMD relapse (p = 0.38). The extent of initial resection (p = 0.42) and the initial ECOG status (p = 0.21) were also not significantly associated with LMD relapse. Of the 21 patients with LMD relapse, 12 had ventricular involvement at diagnosis and 11 surgical entry into the ventricle at the time of initial operation.

**Table 2:** Clinical factors associated with spinal leptomeningeal relapse analysed using chi-square test of independence

	p-value:
Age at initial diagnosis	
<50 vs. ≥ 50 years old	p < 0.01
Initial tumour site	
Temporal lobe vs. other locations	p < 0.01
Ki67	
< 30% vs. ≥ 30%	p = 0.38
Extent of Surgery	
Near-Total vs. Subtotal vs. Biopsy	p = 0.42
MGMT	
Unmethylated vs. Methylated	p < 0.01
ECOG	
0,1 vs. 2,3,4	p = 0. 21

MGMT: O^6^-methylguanine-DNA methyltransferase, ECOG: Eastern Cooperative Oncology Group

## Discussion

This large series of 407 patients treated with initial radical-intent for glioblastoma demonstrates that LMD relapse is an uncommon, but not rare event, occurring in 2.7% of patients at first relapse and 5.1% of patients over the course of their disease following initial aggressive intervention. LMD relapse carried a poor prognosis, associated with a post-relapse median survival of only 39 days. We found that younger patients (< 50 years old), patients with temporal lobe tumours and those lacking MGMT promoter methylation were at increased risk of LMD relapse.

The incidence of LMD relapse in our study is consistent with existing literature. In a single-institution series, Andersen et al. [25] found that 4.6% of their glioma cohort developed LMD. The short survival following LMD relapse is also in line with previous studies, including Li et al. [26] who found that progression with LMD was associated with markedly shorter median time from progression to death (6.0 months) compared to other types of progression (11.5 months). The median overall survival for patients without LMD relapse in our study (17.6 months) is modestly prolonged when compared to historic cohorts [1], which may reflect optimisation of supportive care, second line and subsequent therapies [27, 28]. Autopsy data [29] suggesting that LMD relapse tends to be multifocal along the neuraxis is also supported by our finding that 52% of cases diffusely involved the spine.

We investigated the association between age, tumour site, extent of resection, Ki67 index, IDH1 mutation, MGMT promoter methylation and baseline performance status with subsequent tumour relapse. Factors associated with LMD relapse in this study include younger age, initial temporal lobe tumour location, and unmethylated MGMT promoter status.

Younger age is a recognised risk factor for LMD relapse [7, 26], including in historical autopsy studies [29]. This is in keeping with our finding that the median age of patients with LMD relapse was 48 years compared to 58 years for the cohort as a whole.

In our study, 13 (9.6%) of 136 patients with temporal lobe tumours developed LMD compared to 5.1% of the entire cohort (p < 0.01). This differs from the study by Mandel et al. [16] which did not find an association between the anatomic lobe of the primary tumour and risk of LMD. Other large studies have not addressed this directly [7, 25]. Rare GBMs of specific anatomic locations have been associated with a particularly high risk of LMD. Cerebellar tumours were associated with a 30% risk of LMD at first progression in one study of 17 patients [9]. In their study of pineal GBMs, Niu et al. found that 55% developed LMD. Neither of these rare entities (cerebellar or pineal GBMs) were represented in our cohort. For tumours at such rarer locations, there may be molecular factors at play - for example 55% of the pineal ‘GBMs’ in Niu et al.’s series carried H3 K27M mutations and would no longer be classified as glioblastoma. Another hypothesis for the relationship between tumour location and LMD risk is that proximity to the ventricular system facilitates dissemination in the CSF. Noh et al. [7] demonstrated that tumours in closer proximity to the ventricular system developed LMD at a significantly shorter time interval from diagnosis, but the overall risk of LMD was not affected. Ventricular entry at operation and tumour proximity to the subventricular zone which have also been identified LMD risk factors [12, 13]. Just over half of (12 of 21) of our patients who developed LMD did have involvement of the ventricle at diagnosis and most of these (11 of 12) had the ventricle opened at operation. This potentially supports the hypothesis that one or both of these factors may contribute to the risk of LMD.

We found that of the 12 patients with LMD relapse whose MGMT promoter methylation status was known, all had unmethylated tumours. This result is in contrast to Andersen et al. [25] who found that 25% of the patients in their study who developed LMD had MGMT promoter methylation. Other large series did not address the relationship between MGMT promoter methylation and LMD risk [7, 16]. In their seminal study regarding the impact of glioblastoma MGMT promoter methylation status on patterns of failure, Brandes et al. [30] demonstrated that MGMT promoter methylated tumours had a higher propensity for failure outside the radiotherapy field (42% of cases compared to 15% of unmethylated tumours). Failure outside of the radiotherapy field was also associated with prolonged progression-free and overall survival with one hypothesis being that out-of-field-failure was a reflection of better local control in response to chemoradiotherapy in the methylated tumours. Notably no patient failed with LMD in the Brandes study. LMD failure, which is associated with poor subsequent survival, should be considered an entirely different category to out-of-field intraparenchymal failure. Our finding that unmethylated MGMT promoter status is associated with a greater risk of LMD is therefore consistent with the less favourable biology of this subgroup demonstrated by Brandes et al. and throughout the published literature. We found the association between MGMT promoter methylation status and LMD was strong, no patient with a tumour known to be MGMT methylated had LMD relapse in our study. However these results must be interpreted cautiously as many patients in our study were treated prior to routine testing for MGMT promoter methylation. This data point was therefore missing in 45% of our cohort, introducing substantial risk of bias.

A strength of our study was the large number of consecutive, radically-treated patients in the total cohort (407). A single-institution series this size necessarily included patients diagnosed as early as 2007. There has been substantial progress in our understanding of glioblastoma over this time period, particularly the importance of molecular analysis. A related limitation of the current study therefore is a lack of molecular data particularly pertaining to the earlier patients in the cohort. As discussed above MGMT promoter methylation status was only available for just over half (55%) of patients. We also lacked sufficient data regarding a number of other molecular factors for which previous studies have suggested a possible association with LMD, including BRAF V600E mutation [11, 31], FGFR2 alteration [10], PTEN mutation [32], PIK3CA activating mutation [33], gains at the 1p36 chromosomal region [34] and H3K27M mutation [35]. We also recognise the absolute number of LMD events in our study is relatively small.

Routine craniospinal MRI surveillance postoperatively has been proposed [36, 37] however the low incidence of spinal LMD relapse does not support spinal MRI surveillance. The non-specific symptoms that occur at time of LMD relapse may present a challenge for earlier detection [38]. The factors demonstrated in this study to be associated with LMD relapse may assist the earlier recommendation for utilising spinal MRI in the presence of such symptoms.

There are no current guidelines for the treatment of LMD relapse. In our series, 9 of 21 patients were managed with best supportive care. Palliative systemic therapy was the most commonly utilised anti-cancer treatment (9 of 21 patients), followed by radiotherapy (6 of 21 patients). Data from one series has suggested a numerical improvement in median survival with intrathecal methotrexate compared both other salvage treatments and conservative management, although the difference was only statistically significant with respect to the latter [7]. Prior studies have also shown that radiotherapy may help alleviate LMD symptoms [39, 40]. At relapse re-resection may improve outcomes in select patients [41], but generally only if there is a focal mass causing spinal cord compression. It was not possible due to the small numbers and selection bias to meaningfully determine if any interventions in the current study improved survival. The distribution of relapses as focal or diffuse, and the late timing of relapse after prior salvage therapies means that decision-making for interventions will need to be individualised.

The improving median survival of patients with GBM, and improved control at initial site of disease, will mean that LMD relapse may be experienced more frequently. Of the patients that developed LMD in this study approximately half developed this at second or later relapses with disease already refractory to salvage therapies. Although therapies may need individualisation based on site of disease, new treatment regimens including systemic or intrathecal therapy combined with focal radiation therapy should be prospectively explored given the poor outcome associated with this type of relapse.

## Conclusion

Although uncommon, the frequency of LMD relapse (approximately one in twenty patients in this study) following aggressive multi-modality first line therapy for GBM warrants awareness from clinicians who treat this disease. Younger patients, those with unmethylated tumours, and those with tumours arising from the temporal lobe are at increased risk. Further investigation is required to determine optimal, individualised treatment for patients with LMD relapse.

## Data Availability

The data generated and analysed during the current study is not publicly available due to patient confidentiality but will be available for sharing after local institutional ethics approval. Contact the corresponding author (Dr Patrick Horsley) if needed.
